# Gemcitabine alters sialic acid binding of the glycocalyx and induces inflammatory cytokine production in cultured endothelial cells

**DOI:** 10.1007/s00795-022-00347-4

**Published:** 2023-01-09

**Authors:** Mariko Gunji, Chika Sawa, Minako Akiyama, Shumpei Mukai, Takashi Takaki, Dedong Kang, Kazuho Honda

**Affiliations:** 1 Department of Anatomy, Showa University School of Medicine, 1-5-8 Hatanodai, Shinagawa-ku, Tokyo, 142-8555 Japan; 2 Department of Pathology, Showa University School of Medicine, Tokyo, Japan; 3 Center for Electron Microscopy, Showa University, Tokyo, Japan

**Keywords:** Gemcitabine, Endothelial cell, Glycocalyx, Sialic acid, Platelet–endothelial cell adhesion molecule (PECAM), Vascular endothelial growth factor receptor 2 (VEGFR2), Interleukin-1β, Interleukin-6

## Abstract

Gemcitabine (GEM) is an anticancer drug inhibiting DNA synthesis. Glomerular thrombotic microangiopathy (TMA) has been reported as an adverse effect. However, the precise mechanism of GEM-induced endothelial injury remains unknown. Cultured human umbilical vein endothelial cells (HUVECs) in the confluent phase were exposed to GEM (5–100 μM) for 48 h and evaluated cell viability and morphology, lectin binding concerning sialic acid of endothelial glycocalyx (GCX), and immunofluorescent staining of platelet–endothelial cell adhesion molecule (PECAM) and vascular endothelial growth factor receptor 2 (VEGFR2). The mRNA expression of α2,6-sialyltransferase (ST6Gal1), sialidase (neuraminidase-1: NEU-1), and interleukin (IL)-1β and IL-6 was also evaluated. GEM exposure at 5 μM induced cellular shrinkage and intercellular dissociation, accompanied by slight attenuation of PECAM and VEGFR2 immunostaining, although cell viability was still preserved. At this concentration, lectin binding showed a reduction of terminal sialic acids in endothelial GCX, probably associated with reduced ST6Gal1 mRNA expression. IL-1β and IL-6 mRNA expression was significantly increased after GEM exposure. GEM reduced terminal sialic acids in endothelial GCX through mRNA suppression of ST6Gal1 and induced inflammatory cytokine production in HUVECs. This phenomenon could be associated with the mechanism of GEM-induced TMA.

**Supplementary Information:**

The online version contains supplementary material available at 10.1007/s00795-022-00347-4.

## Introduction

Gemcitabine (GEM) is an analog of a standard nucleotide component, deoxycytidine triphosphate (dCTP). It inhibits normal DNA synthesis, whereas it produces abnormal DNA sequence, which leads to apoptosis of tumor cells [1]. It has been introduced clinically as an anticancer drug for many malignant neoplasms derived from the pancreas, lung, breast, and biliary and urinary tracts [2]. Its metabolite induces indirect inhibition of DNA synthesis by reducing the concentration of dCTP via inhibition of ribonucleotide reductase (RR), which synthesizes dCTP [1]. Figure S-1 shows the details of the intracellular metabolism of GEM.

Clinically, kidney injury presenting hemolytic uremic syndrome (HUS) is one of the major adverse effects of GEM [3, 4]. Pathology of the kidney biopsies of the patients with GEM-induced HUS has been reported as glomerular thrombotic microangiopathy (TMA) [5–8]. Morphological evidence suggests that endothelial injury could be an etiology of the glomerular TMA induced by GEM, although its precise mechanism has not been elucidated yet [9].

**Fig. 1:**
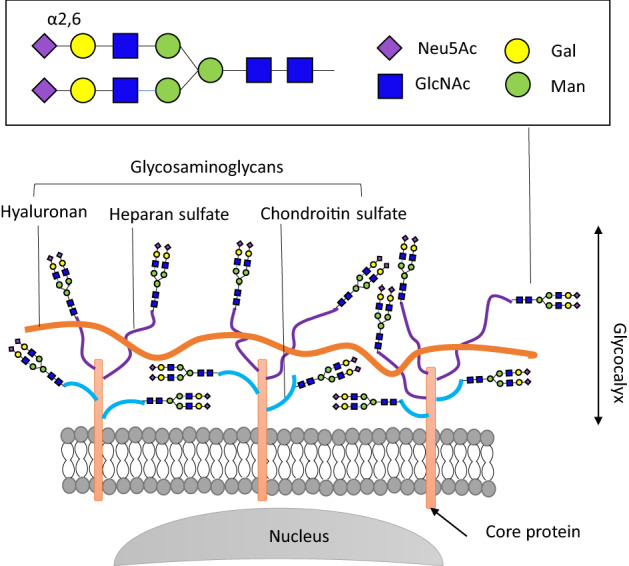
Structure and components of human endothelial glycocalyx (GCX). GCX is present on the cell surface of endothelial cells. Glycosaminoglycans (GAGs) such as hyaluronic acid, heparan sulfate, and chondroitin sulfate are attached to the core proteins of GCX. In humans, sialic acid (Neu5AAC) is terminally bound to α-2,6-galactose (Gal) of the sugar chains of these GAGs. Neu5Ac, N-acetyl-neuraminic acid; Gal, galactose; GlcNAc, N-acetyl-D-glucosamine; Man, mannose

Endothelial cells are associated with many physiological and pathological phenomena, and a structure covering the endothelial surface, called endothelial glycocalyx (GCX), has attracted scientific interest [10, 11]. Schematic structure of the endothelial GCX is shown in Fig. 1 [10]. It is a thin layer composed of various proteins and carbohydrates, core proteins binding to the cell membrane (syndecan, glypican, etc.), glycosaminoglycan (GAG) binding to the core proteins (hepa sulfate, chondroitin sulfate, etc.) and hyaluronic acid, and free long polysaccharide chains. Endothelial GCX is thought to be associated with the regulation of intercellular adhesion [12], protection from infectious microorganisms in sepsis [13, 14], endothelial transport [10], and signal transduction induced by shear stress [15]. It is also associated with proinflammatory, procoagulant, and proliferative reactions in endothelial cells [10]. Alterations of the endothelial GCX were studied in animal models of sepsis [16, 17], acute stress of surgery [18], trauma [19], and diabetic nephropathy [20, 21].

Due to its delicate and fragile components, morphological detection of GCX was difficult, and this situation interfered with the thorough investigation of the morphology and function of the endothelial GCX [22]. Recently, new technologies using lectin histochemistry and electron microscopy have been introduced to elucidate its alterations in various physiological and pathological conditions [23, 24].

Sialic acid is usually present at the terminal of the polysaccharide chains of GAGs and plays important roles in regulating cellular functions (Fig. 1). The binding site of sialic acid determines the status of various biological phenomena, including intercellular adherence and interaction, cell-to-matrix interaction, and affinity of virus receptors [25]. The terminal sialic acid is synthesized by sialyltransferase, comprising over 20 enzymes in mammals, and is categorized into four families according to the type of carbohydrate linkage [26]. Among these four families, the α2,6-sialyltransferase family (ST6Gal1 and ST6Gal2) are present in endothelial cells and synthesize the terminal sialic acids of endothelial GCX. Furthermore, mRNA expression analysis revealed that the *ST6GAL1* gene is expressed in various organs, but the *ST6GAL2* gene is expressed only in the embryonic brain. *ST6GAL1* gene knockout mice demonstrated abnormal endothelial hyperpermeability due to loss of sialic acids on endothelial PECAM (CD31) molecule and reduction of PECAM hemophilic interaction at the intercellular adherent junction [12, 27, 28]. The impaired function of PECAM–VEGFR2–β3 integrin complex is also observed in the *ST6GAL1* gene knockout mice, which regulates several important endothelial functions such as angiogenesis and apoptosis [12, 27, 28].

To this background, we investigated alterations of cultured HUVECs after GEM exposure, especially focusing on the sialic acid of GCX to explore the mechanism of GEM-induced endothelial injury. Moreover, the significant roles of sialic acids in endothelial GCX are discussed for several important functions in vascular endothelial cells.

## Materials and methods

### Materials

GEM (gemcitabine hydrochloride, G0367, Tokyo Chemical Industry Co., Ltd.) was dissolved in PBS (-) at 100 mM and stored at -30℃. Type I collagen (Atelocollagen Acidic Solution IPC-30, KOKEN, Japan) was diluted with 0.01-N HCl at 0.01% and stored at 4℃.

### Cell culture

Human umbilical vein endothelial cell (HUVEC, single donor; PromoCell, Germany) was routinely cultured in an endothelial cell growth medium two kit (PromoCell, Germany) on 0.1% collagen coat dish (IWAKI, Japan) at 37℃ under 5% CO_2_. For all experiments, cells were used between three and six passages. For coating dishes and cover glass (Matsunami, Japan) with type I collagen, the plate was soaked in 0.01% collagen for 2 h at room temperature, followed by five washes with sterile water.

### Cell viability assay

To assess cell viability at both cell growth and confluent phase, an MTT assay was conducted. For the growth phase, HUVECs were seeded in collagen-coated 96-well plate at 5 × 10^3^ cells/well, and various concentrations (0–0.5 μM) of GEM were added on the following day and incubated for a further 48 h. For the confluent phase, HUVECs were seeded at 2 × 10^3^/well and incubated for 5 days. Subsequently, various concentrations (0–100 μM) of GEM were added and incubated for a further 48 h. To assess cell viability, CellTiter 96^®^ Non-Radioactive Cell Proliferation Assay (Promega, USA) was used according to the manufacturer’s instructions. Briefly, cells were washed with Dulbecco’s Modified Eagle Medium (DMEM; Corning, USA without phenol red) three times, and 100 ul of DMEM (without phenol red) and 15 μl of 3-[4,5-dimethylthiazol-2-yl]-2,5 diphenyl tetrazolium bromide (MTT) reagent were added. After incubation at 37℃ for 4 h, 100 ul of stop reagent was added, and optical density was measured using a multi-plate reader (FLUOsar Omega, BMG LABTECH, Germany) at a wavelength of 570 nm.

### Lectin staining

1.2×10^4^/well HUVECs were seeded on 12-well plates containing collagen-coated cover glass and incubated for 5 days to allow confluence. GEM was then added to the medium, and cells were incubated for further 2 days before immunocytochemistry. To detect several forms of glycoprotein, the following lectins were used: fluorescein isothiocyanate (FITC)-linked wheat germ agglutinin (WGA-FITC) for N-acetyl-D-glucosamine and sialic acid residues; FITC-conjugated Sambucus nigra lectin (SNA-FITC) for preferentially sialic acid attached to terminal galactose in α2,6 and, to a lesser degree, α2,3 linkage; and FITC-conjugated Ricinus communis agglutinin I (RCA-I-FITC) for desialylation of galactose and N-acetyl-D-galactosamine. We compared the binding of SNA to detect sialic acid and the binding of RCA-1 to detect desialylated galactose or N-acetyl-D-galactosamine, which was reported as a useful method to analyze terminal sialylation of N-glycan of immunoglobulin G [29]. HUVECs on the cover glass were stained as follows: Cells were washed with serum-free medium once before each lectin staining step. Each lectin was applied to cells for 30 min at 37 ℃ at a dilution rate as follows: FITC-WGA (1:500) and FITC-SNA (1:200) and FITC-RCA-I (1:1000). Negative control staining was performed using PBS without each lectin. Hoechst 33,342 (1:300) was added to live cells for the last 3 min and subsequently, cells were washed three times with PBS (+) (phosphate-buffered saline with 1 mM CaCl_2_ and 1 mM MgCl_2_) and following 4% PFA (+) (paraformaldehyde phosphate buffer solution with 1 mM CaCl_2_ and 1 mM MgCl_2_) for 8 min. Finally, the cover glass-attached stained cells were transferred onto a glass slide and encapsulant with Aqueous Mounting Medium PermaFluor (Thermo Scientific).

### Immunocytochemistry

For PECAM (CD31), cells were washed with serum (−) medium once and stained with Hoechst 33342 (1:250 dilution) for 3 min and subsequently fixed with 4% PFA ( +) for 8 min. After washing with PBS ( +) three times and blocking with 5% skim milk (Nacalai, Japan) for 30 min, anti-human CD31 (Clone JC70A, M0823, DAKO; 1:40 dilution) was incubated for 30 min at room temperature. Cells were washed with PBS ( +) three times, and goat anti-mouse IgG (H + L) highly cross-adsorbed secondary antibody conjugated with Alexa Fluor 546 (A11030, Invitrogen) was incubated for 30 min at room temperature.

For ST6Gal1, cells were fixed as the CD31 staining and subsequently permeabilized with 0.1% Triton X for 5 min and 5% goat serum (S-1000, Vector Laboratories) for 1 h at room temperature. Anti-α2,6-sialyltransferase (M2) antibody (28047, IBL; 1:100 dilution) was then incubated for 45 min at room temperature and after being washed with PBS ( +) three times, goat anti-rabbit IgG (H + L) highly cross-adsorbed secondary antibody conjugated with Alexa Fluor Plus 488 (A32731, Thermo Fisher 1:100 dilution) was added for 45 min, and further 4',6-diamidino-2-phenylindole, dihydrochloride (DAPI, Dojin, Japan) was added for the last 3 min.

For NEU-1/sialidase-1, cells were stained with Hoechst 33,342, fixed, and permeabilized as the ST6Gal1 staining. After being blocked with 3% BSA for 1 h, cells were incubated with anti-NEU-1/sialidase-1 (NBP1-87755, Novusbio; 1:4000 dilution) for 1 h at room temperature. Subsequently, the cells were washed with PBS ( +) three times and the goat anti-rabbit IgG (H + L) highly cross-adsorbed secondary antibody conjugated with Alexa Fluor Plus 488 (A32731, Thermo Fisher) (1:100 dilution) was added for 1 h.

For VEGFR2, cells were fixed as the CD31 staining. After washing with PBS ( +) three times, cells were blocked with 1% BSA containing 0.1% polyoxyethelenesorbitan monolaurate (Tween 20) for 1 h, followed by incubation with anti-VEGF receptor 2 antibody (Abcam, ab39638 1:1000 dilution) overnight at 4℃. Subsequently, cells were washed with PBS (+) three times, and the goat anti-rabbit IgG (H + L) highly cross-adsorbed secondary antibody conjugated with Alexa Fluor Plus 488 (A32731, Thermo Fisher 1:500 dilution) for 1 h and further 4',6-diamidino-2-phenylindole, dihydrochloride (DAPI, Dojin, Japan) was added for the last 3 min. In each immunocytochemical staining, negative control staining was performed using PBS without each primary antibody.

### Confocal laser-scanning fluorescence microscopy

Confocal laser-scanning fluorescence microscopy images were captured with a NIKON A1 inverted confocal microscope system (Nikon, Japan). It is equipped with a 60 × oil immersion objective, and 488, 561, and 405 nm were used as excitation sources for the fluorophores stained in the sample. Black level (background offset) was adjusted to eliminate autofluoresence from unstained cells.

### RNA extraction and reverse transcription polymerase reaction

Total RNA was extracted from HUVECs using Sepasol-RNA I Super G (Nacalai, Japan), and the quality was validated spectrophotometrically. First-strand cDNA was synthesized with an AffinityScript QPCR cDNA Synthesis Kit (Stratagene) according to the protocol provided by the manufacturer, using 300 ng total RNA. Subsequently, PCR was conducted using EmeraldAmp PCR Master Mix (Takara, Japan). A commonly used gene, namely, *GAPDH*, was used for RT-PCR, and gene-specific primers for GAPDH, VEGFR1, VEGFR2, ST6Gal1, and sialidase are shown in the supplementary material (Table S1). Agarose electrophoresis was conducted, and the gels were stained with ethidium bromide and analyzed with ChemiDoc XRS + imaging system (Bio-Rad).

### Statistical analysis

For each treatment, triplicate measurements were made. Using Microsoft Excel, the data were analyzed by one-way analysis of variance. Values are expressed as mean ± standard deviation. The t-test was used to calculate statistical significance, and a *P* < 0.05 was considered statistically significant.

## Results

### Effects of GEM on cell viability during growth and confluent phases in HUVECs

**Fig. 2:**
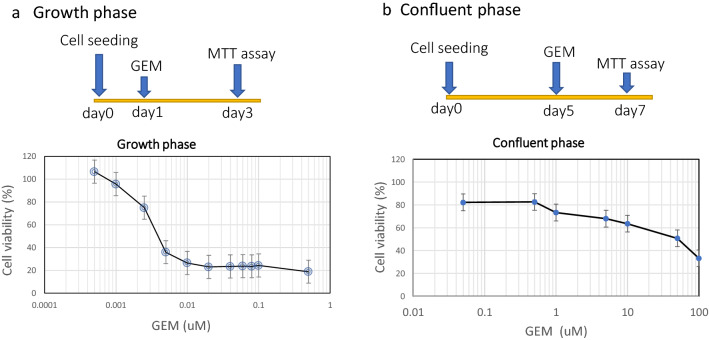
Effect of GEM on cell viability of HUVECs in growth and confluent phases. **a** Cell viability assay in the growth phase. GEM in various concentrations ranging from 0.025 to 0.5 μM was added to the culture medium on the first day after cell seeding, and an MTT assay was conducted on the third day (48 h later). The half-maximal (50%) inhibitory concentration (IC50) of cell viability was 0.025–0.05 μM in the growth phase. **b** Cell viability assay in the confluent phase. GEM at various concentrations ranging from 0.05 to 100 μM was added to the culture medium on the fifth day after cell seeding, and an MTT assay was conducted on the seventh day (48 h later). The IC50 of cell viability was 50–100 μM in the confluent phase. The GEM sensitivity of HUVECs was approximately 1/1,000 to 1/4,000 in the confluent phase compared to the growth phase

Figure 2 shows the relationship between GEM concentration and cell viability during growth and confluent phases in HUVECs. The half-maximal (50%) inhibitory concentration (IC50) of cell viability was 0.025–0.05 μM in the growth phase (Fig. 2-a) and 50–100 μM in the confluent phase (Fig. 2-b). The GEM sensitivity to cell viability suppression was lower in the confluent phase (about 1/1,000–1/4,000) than in the growth phase.

### Alterations of cellular morphology and PECAM (CD31) and VEGFR2 expression in HUVECs after GEM exposure (Fig. 3)

**Fig. 3:**
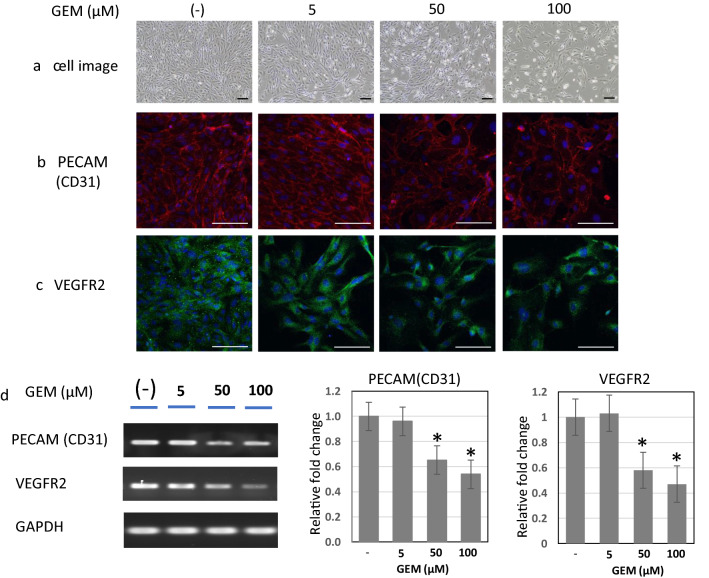
Cellular morphology and PECAM (CD31) and VEGFR expressions in HUVECs after GEM exposure. a Cellular morphology of HUVECs after GEM exposure in the confluent phase. The cellular morphology showed no significant alterations at 5 μM but began showing a tendency of oval- or spindle-shaped shrinkage and intercellular widening at 50 μM; this tendency became evident in 100 μM of GEM concentration. (× 20). Scale bars: 100 μm. b Immunofluorescent expression of PECAM in HUVECs after GEM exposure CD31. PECAM is expressed onto the plasma membrane in the control state. GEM exposure at 5 µM slightly attenuated the PECAM expression, and it became weaker at 50 µM. The lining of the positivity on the cell membrane became irregular and disrupted at 100 µM [Red: PECAM, Blue: DAPI (nuclei)]. Scale bars: 100 μm. c Immunofluorescent expression of VEGFR2 in HUVECs after GEM exposure. VEGFR2 is mainly present in the cytoplasm in the control state, and it slightly attenuated after GEM exposure at 5 µM and became more scattered as the concentration of GEM increased from 50 µM to 100 µM. [Green: VEGFR2, Blue: DAPI (nucleus)]. Scale bars: 100 μm. d PECAM and VEGFR2 mRNA expression in HUVECs after GEM exposure (RT-PCR). PECAM and VEGFR2 mRNA expression were both detected in HUVECs by PT-PCR. After GEM exposure, the PECAM mRNA expression did not remarkably change at 5 μM but decreased at 50 μM and 100 μM. The VEGRF2 mRNA expression also did not remarkably change at 5 μM but reduced at 50 μM and 100 μM

#### Cellular morphology of HUVECs after GEM exposure in confluent phase (Fig. 3-a)

On the fifth day after seeding, HUVECs in the confluent phase were exposed to GEM at various concentrations (0, 5, 50, and 100 μM) and cultured for 48 h. Figure 3-a presents cellular appearances at multiple concentrations. The cell image showed no significant alterations at 5 μM, but tended to show oval or spindle-shaped shrinkage and intercellular widening at 50 μM, which became evident in 100 μM of GEM. Due to the widening of intercellular spaces, cell density seemed to decrease at 100 μM (Fig. 3-a).

#### Immunofluorescent expression of PECAM and VEGFR2 in HUVECs after GEM exposure (Fig. 3-b, 3-c)

In the control state without GEM exposure, PECAM was expressed on plasma membranes. GEM exposure at 5 µM slightly attenuated the PECAM expression on the plasma membrane; it became weaker at 50 µM and the lining of PECAM positivity on the cell membranes became irregular and disrupted in 100 µM (Fig. 3-b). On the other hand, VEGFR2 is mainly expressed in the cytoplasm of HUVECs without GEM exposure. The cytoplasmic expression of VEGFR2 was slightly reduced after GEM exposure in 5 µM and became more scattered as the GEM concentration increased from 50 µM to 100 µM.

#### PECAM and VEGFR2 mRNA expression in HUVECs after GEM exposure (Fig. 3-d)

Both PECAM and VEGFR2 mRNA expression were detected in HUVECs by RT-PCR in the control state without GEM exposure. After 48 h of exposure to GEM at different concentrations, PECAM mRNA expression did not significantly change at 5 μM but decreased at 50 μM and 100 μM. The VEGRF2 mRNA expression did not change at 5 μM but decreased at 50 μM and 100 μM as well (Fig. 3-d). These results were generally consistent with the results of immunofluorescence staining of PECAM and VEGFR2.

### Alterations of lectin binding and sialic acid-related enzyme expression in HUVECs after GEM exposure (Fig. 4)

**Fig. 4:**
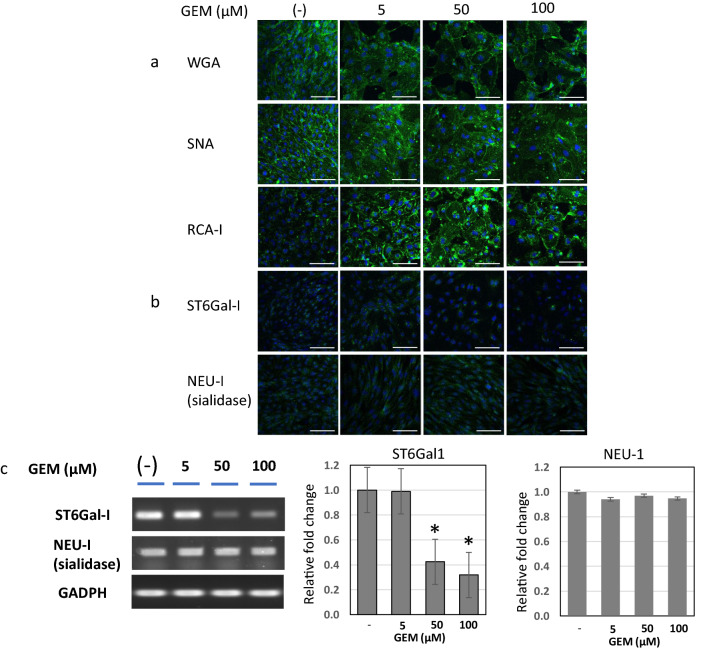
Lectin binding and sialic acid-related enzyme expression in HUVECs after GEM exposure. a Lectin binding of endothelial GCX in HUVECs after GEM exposure. WGA: Exposure to GEM at 5, 50, and 100 μM concentrations did not change WGA-lectin binding notably compared with the control state. SNA: SNA lectin binding was slightly reduced in 5 μM and further diminished in 50 μM and 100 μM. RCA-I: RCA-I binding was sparsely present on the cell membrane in the control state and significantly increased after GEM exposure at 5 μM and further augmented at 50 and 100 μM. [Green: WGA, SNA, RCA-1, ST6Gal1 and NEU-1, Blue: Hoechst33342 (nuclei)]. Scale bars: 100 μm. b Immunofluorescent staining of sialic acid-related enzyme in HUVECs after GEM exposure. ST6Gal1: In the control state, cytoplasmic localization of ST6Gal1 was detected as a granular dot, corresponding to the Golgi apparatus. The immunofluorescent staining of ST6Gal1 did not significantly change at 5 μM but significantly attenuated in 50 uM and 100 uM of GEM. NEU-1 (sialidase): NEU-1 was weakly present in the cytoplasm in the control state, and the staining did not significantly change after GEM exposure at 5, 50, and 100 μM. Scale bars: 100 μm. c mRNA expression of sialic acid-related enzymes after GEM exposure (RT-PCR). ST6Gal1 mRNA expression was detected in the control state and did not change at 5 uM GEM but decreased at 50 and 100 uM of GEM, whereas NEU-1 (sialidase) mRNA expression was stable and maintained similarly to the control state even after GEM exposure in any concentration

#### Lectin binding of endothelial GCX in HUVECs after GEM exposure (Fig. 4-a)

##### Wheat germ agglutinin (WGA)

WGA is a lectin that binds to N-acetyl-D-glucosamine and sialic acid of endothelial GCX. Exposure to GEM at 5, 50, and 100 μM concentrations did not alter WGA-lectin binding significantly compared with the control state without GEM exposure (Fig. 4-a).

##### Nigra agglutinin (SNA)

SNA is a lectin that binds to the terminal sialic acids of sugar chains in endothelial GCX. Exposure of GEM in 5 μM slightly reduced the SNA lectin binding, which was further diminished in 50 μM and 100 μM. These results showed that GEM exposure attenuated the terminal sialic acids bound to α2,6-terminal galactose of endothelial GCX (Fig. 4-a).

##### Ricinus communis agglutinin I (RCA-I) 

In contrast to SNA, RCA-I is a lectin that binds to desialylated galactose in the endothelial GCX. RCA-I binding was sparsely detected on the cell membranes of HUVECs in the control state. However, it notably increased after GEM exposure at 5 μM and further augmented at 50 and 100 μM (Fig. 4-a). This reflected the reduction of terminal sialic acids and consequent baring of galactose in the sugar chains of endothelial GCX after GEM exposure.

#### Immunofluorescent staining of sialic acid-related enzymes in HUVECs after GEM exposure: ST6Gal1 and sialidase (NEU-1) (Fig. 4-b)

##### Immunofluorescent staining of ST6Gal1

ST6Gal1 is an enzyme that adds sialic acid at α-2,6-galactose of the sugar chains in the Golgi apparatus of endothelial cells. In the control state of HUVECs, cytoplasmic localization of ST6Gal1 was detected as a granular dot corresponding to the Golgi apparatus. The immunofluorescent staining of ST6Gal1 did not notably change significantly after GEM exposure at 5 μM, but was significantly attenuated after GEM exposure at 50 uM and 100 uM concentrations (Fig. 4-a).

##### Immunofluorescence staining of sialidase (neuraminidase-1: NEU-1) 

Sialidase, i.e., neuraminidase-1 (NEU-1), is an enzyme that breaks down the terminal sialic acids from the galactose of GCX sugar chains. In the control state without GEM exposure, NEU-1 was weakly present as a fine-granular appearance in the cytoplasm. The immunofluorescent staining of NEU-1 did not notably change after GEM exposure at 5, 50, and 100 μM (Fig. 4-a).

#### mRNA expression of sialic acid-related enzymes after GEM exposure: ST6Gal1 and sialidase (NEU-1) (Fig. 4-c)

The mRNA expression of ST6Gal1 and NEU-1 was detected in the control state of HUVECs. The ST6Gal1 mRNA expression did not change after GEM exposure in 5 µM, but decreased after GEM exposure in 50 µM and 100 µM. Nonetheless, the NEU-1 mRNA expression was stable and maintained similarly after GEM exposure at all concentrations compared with the control state (Fig. 4-c).

### Alterations of inflammatory cytokine (IL-6, IL-1β) mRNA expression after GEM exposure(Fig. 5) 

**Fig. 5:**
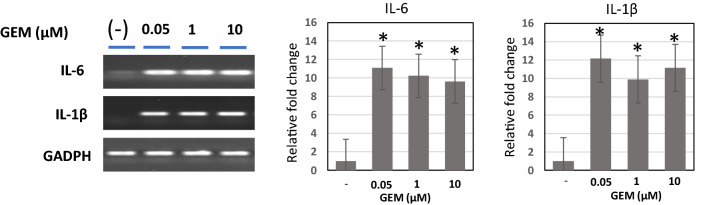
Inflammatory cytokine mRNA expression in HUVECs after GEM exposure. IL-6 and IL-1β mRNA expression were barely detectable in the control state; however, after the exposure to GEM, remarkable increases in IL-6 and IL-1β mRNA expression were observed in 5 μM, and similar increases were still detected in both 50 and 100 μM

In the control state of HUVECs, IL-6 and IL-1β mRNA expression were barely detectable. After the exposure to GEM, remarkable increases in IL-6 and IL-1β mRNA expression were observed at 5 μM, and significant increases were similarly detected at 50 and 100 μM GEM concentration (Fig. 5).

## Discussion

### Reduction of terminal sialic acid of GCX and endothelial injury

This study revealed that GEM decreased the terminal sialic acid of endothelial GCX, which was demonstrated by decreased binding of SNA lectin to detect sialic acid and increased binding of RCA-1 lectin to detect the denudation of sialic acid. We speculated that this sialic acid reduction may not be caused by the increase in NEU-1 (sialidase), a breakdown enzyme of sialic acid, but by the decrease in ST6Gal1, a major sialic acid transferase at the terminal galactose of GCX sugar chains.

Terminal sialic acids bound to various glycoproteins of GCX play a vital role in regulating physiological and pathological processes, including embryogenesis, infection, inflammatory disease, and cancer [30, 31]. In endothelial cells, sialic acids are conjugated with the glycoproteins localized in cell membranes and regulate many endothelial functions, such as vascular permeability and angiogenesis [32, 33]. Kitazume et al. revealed that α2,6 sialic acid on PECAM played vital roles in the hemophilic interaction of PECAM and downstream anti-apoptotic signaling in endothelial cells [12, 27]. Lee C et al. also revealed a significant role of the sialylation state of PECAM in angiogenesis, demonstrating that desialylation of PECAM by NEU-1 (sialidase) impaired capillary-like tube formation (angiogenesis) in human lung microvascular endothelial cells [34].

Furthermore, PECAM forms a mechanosensory membrane complex with VEGFR2 and VE-cadherin molecules, and their cooperation regulates many endothelial functions [12, 27]. VEGFR2 is a highly N-glycosylated receptor tyrosine kinase and is involved in the pro-angiogenic signaling of VEGF. Chiodelli P et al. demonstrated that 2,6-sialylated glycan of VEGFR2 is relevant in the binding of VEGF-A and pretreatment with SNA lectin to mask this sialylation or NEU-1 to denudate the sialic acids reduced VEGF-A binding, and subsequently disturbed VEGFR2 signaling, which impaired the process of angiogenesis [35]. Similarly, Chandler KB et al. reported that the terminal sialic acids capping the N-glycan at Asn 247 specifically regulated the ligand-dependent activation and signaling of VEGFR2 in endothelial cells [36].

In addition to the reduction of terminal sialic acids of endothelial glycoprotein molecules, GEM directly reduced PECAM- and VEGFR2-mRNA expression and also their protein expression by immunofluorescent examination. The reduced amounts of PECAM and VEGFR2 molecules in endothelial membranes could lead to impaired endothelial functions associated with the PECAM–VEGFR2 complex and its signaling transduction [12, 37]. These results suggest that the significant functional roles of sialic acid in vascular physiology and the disruption of sialic acid in endothelial GCX could lead to severe endothelial dysfunction and injury.

Moreover, we revealed the suppression of ST6Gal1 mRNA and the subsequent decrease of sialic acid expression in the endothelial cell membrane after GEM exposure. Recent studies indicated that ST6Gal1 is a crucial enzyme in the sialylation of N-glycans and plays essential roles in the immune system, pathogen recognition, and cancer biology [31, 38]. ST6Gal1 is also detected in the tumor vessels and regulates tumor angiogenesis through PECAM–VEGFR2 complex signaling, which is also indirectly associated with tumor progression or regression [28, 38, 39]. Therefore, the expression of ST6Gal1 in endothelial cells is important for vascular physiology and angiogenesis and the reduction of ST6Gal1 mRNA expression by GEM could be associated with endothelial injury leading to TMA.

### Cytokine production in endothelial cells and its clinical implications

This study also demonstrated marked inflammatory cytokine production after GEM exposure in HUVECs. Cytokine production by endothelial cells has been recognized in various clinical situations. In sepsis, endothelial cells covering the vascular luminal surface of all organs are triggered to stimulate by various exogenous pathogens, microbial toxins, or endogenous danger signals via pattern recognition receptors (PRRs), such as pathogen-associated molecular patterns (PAMPs), damage-associated molecular patterns (DAMPs), and toll-like receptors (TLRs). These stimulations drive endothelial cells toward proinflammatory, proapoptotic, and procoagulant phenotypes [11]. IL-6 is one of the major cytokines produced by endothelial cells and inflammatory cells in sepsis, cytokine release syndrome associated with chimeric-antigen receptor T-cell therapy [40], and cytokine storm syndrome after COVID-19 infection [41]. Several experimental studies demonstrated IL-6 production by cultured endothelial cells after stimulation by LPS [42] and autoantibodies from patients with scleroderma renal crisis [43].

Kang S et al. performed clinical investigations of patients with sepsis, acute respiratory distress syndrome, burns, and COVID-19 infection and demonstrated elevated levels of four cytokines: IL-6, IL-8, monocyte chemotactic protein-1 (MCP-1) and IL-10, and plasminogen activator inhibitor-1 (PAI-1) in serum [44]. They also revealed by in vitro experiment using HUVECs that the production of these cytokines, including IL-6 and PAI-1, was increased after TNFα or IL-1β stimulation. Considering these clinical and experimental results, they speculated an essential role of IL-6 signaling in endothelial dysfunction in sepsis and cytokine release syndrome [44]. Furthermore, McConnell MJ et al. revealed significant elevations of serum IL-6 as well as several biomarkers of coagulopathy and endotheliopathy, such as factor VIII, fibrinogen, d-dimer, and von Willebrand factor (vWF) in 43 COVID-19 patients with liver injury, and demonstrated the histological expression of these coagulation factors in liver sinusoidal endothelial cells (LSECs) in the biopsy specimens [45]. They also revealed the cultured LSECs stimulated by IL-6 produced proinflammatory cytokines through JAK1-STAT1/3 signaling [45].

Krick S et al. recently reported that knockdown of the ST6GAL1 gene or inhibition of beta-site amyloid precursor protein-cleaving enzyme 1 (BACE-1), a synthetic enzyme of ST6Gal1, reduced sialic acid expression and elevated mRNA expression and secretion of IL-6 in cultured human bronchial epithelial cells (HBECs) [46]. They speculated that the sialic acids in bronchial membranes are essential in maintaining bronchial physiology and dysfunction and the disruption of sialic acids by cigarette smoking or chronic obstructive pulmonary diseases could induce and sustain chronic inflammatory reactions through IL-6 production and secretion [46]. These results could support our hypothesis that GEM causes the disruption of sialic acid in endothelial GCX through inhibition of ST6Gal1 expression and triggers cytokine production from endothelial cells that facilitate endothelial injury leading to TMA.

## Conclusions

This in vitro study revealed that GEM caused the disturbance of intercellular adherence, which was probably associated with reduced amounts of terminal sialic acid in endothelial GCX in HUVECs. This reduction of terminal sialic acid was derived from the inhibition of ST6Gal1 expression and stimulation of cytokine production, such as IL-6 and IL-1β. These phenomena may be associated with the pathogenesis of GEM-induced glomerular TMA. Further investigations are required to confirm this anticipating hypothesis, i.e., disturbed sialylation of endothelial GCX induces endothelial dysfunction through proinflammatory stimulation pathway and leads to subsequent endothelial injury.

## Supplementary Information

Below is the link to the electronic supplementary material.

Supplementary file1 Figure S-1：Intracellular metabolic pathway of GEM and its associated enzymes After being up-taken into cells by equilibrative nucleoside transporter 1 (ENT1) and ENT2, GEM [2,2-difluorodeoxycytidine (dFdC)] is phosphorylated by deoxycytidine (dCK) into active nucleotide diphosphate (dFdCDP) and triphosphate (dFdCTP). These active nucleotides exhibit cell-killing activity by directly and indirectly inhibiting DNA synthesis. Directly, dFdCTP causes cell death (or apoptosis) after being incorporated into DNA strands by DNA polymerase in competition with deoxycytidine triphosphate (dCTP). Indirectly, dFdCDP inhibits RR (ribonucleotide reductase) to reduce intracellular dCTP concentration, thus indirectly enhancing DNA synthesis inhibition. dFdC, 2,2-difluorodeoxycytidine; ENT1 and ENT2, equilibrative nucleoside transporter 1 and 2; dCDA, deoxycytidine deaminase; dFdU, difluorodeoxyuridine; dCK, deoxycytidine kinase; 5’-NT, 5’-nucledtidase; dFdCMP, gemcitabine monophosphate; dCMPK, dCMP deaminase; dFdUMP, 2’2’-difluorodeoxyuridine monophosphate; dFdCDP, gemcitabine diphosphate; RR, ribonucleotide reductase; TS, thymidylate synthase; NDPK, nucleoside diphosphate kinase; dFdCTP, gemcitabine triphosphate (PPTX 40 KB)

## References

[1] Mini E, Nobili S, Caciagli B, Landini I, Mazzei T (2006). Cellular pharmacology of gemcitabine. Ann Oncol.

[2] Toschi L, Finocchiaro G, Bartolini S, Gioia V, Cappuzzo F (2005). Role of gemcitabine in cancer therapy. Future Oncol.

[3] Fung MC, Storniolo AM, Nguyen B, Arning M, Brookfield W, Vigil J (1999). A review of hemolytic uremic syndrome in patients treated with gemcitabine therapy. Cancer.

[4] Perazella MA (2012). Onco-nephrology: renal toxicities of chemotherapeutic agents. Clin J Am Soc Nephrol.

[5] Izzedine H, Isnard-Bagnis C, Launay-Vacher V, Mercadal L, Tostivint I, Rixe O, Brocheriou I, Bourry E, Karie S, Saeb S, Casimir N, Billemont B, Deray G (2006). Gemcitabine-induced thrombotic microangiopathy: a systematic review. Nephrol Dial Transplant.

[6] Blake-Haskins JA, Lechleider RJ, Kreitman RJ (2011). Thrombotic microangiopathy with targeted cancer agents. Clin Cancer Res.

[7] Humphreys BD, Sharman JP, Henderson JM, Clark JW, Marks PW, Rennke HG, Zhu AX, Magee CC (2004). Gemcitabine-associated thrombotic microangiopathy. Cancer.

[8] Glezerman I, Kris MG, Miller V, Seshan S, Flombaum CD (2009). Gemcitabine nephrotoxicity and hemolytic uremic syndrome: report of 29 cases from a single institution. Clin Nephrol.

[9] Brocklebank V, Wood KM, Kavanagh D (2018). Thrombotic microangiopathy and the kidney. Clin J Am Soc Nephrol.

[10] Reitsma S, Slaaf DW, Vink H, ZandvoortMAMJv, EgbrinkMGAo, (2007). The endothelial glycocalyx: composition, functions, and visualization. Pflugers Arch.

[11] Joffre J, Hellman J, Ince C, Ait-Oufella H (2020). Endothelial responses in sepsis. Am J Respir Crit Care Med.

[12] Kitazume S, Imamaki R, Ogawa K, Taniguchi N (2014). Sweet role of platelet endothelial cell adhesion molecule in understanding angiogenesis. Glycobiology.

[13] Uchimido R, Schmidt EP, Shapiro NI (2019). The glycocalyx: a novel diagnostic and therapeutic target in sepsis. Crit Care.

[14] Song JW, Zullo J, Lipphardt M, Dragovich M, Zhang FX, Fu B, Goligorsky MS (2018). Endothelial glycocalyx-the battleground for complications of sepsis and kidney injury. Nephrol Dial Transplant.

[15] Lupu F, Kinasewitz G, Dormer K (2020). The role of endothelial shear stress on haemodynamics, inflammation, coagulation and glycocalyx during sepsis. J Cell Mol Med.

[16] Schmidt EP, Yang Y, Janssen WJ, Gandjeva A, Perez MJ, Barthel L, Zemans RL, Bowman JC, Koyanagi DE, Yunt ZX, Smith LP, Cheng SS, Overdier KH, Thompson KR, Geraci MW, IDouglas IS, Pearse DB, Tuder RM, (2012). The pulmonary endothelial glycocalyx regulates neutrophil adhesion and lung injury during experimental sepsis. Nat Med.

[17] Watanabe E, Akamatsu T, Ohmori M, Kato M, Takeuchi N, Ishiwada N, Nishimura R, Hishiki H, Fujimura L, Ito C, Hatano M (2022). Recombinant thrombomodulin attenuates hyper-inflammation and glycocalyx damage in a murine model of Streptococcus pneumoniae-induced sepsis. Cytokine.

[18] Astapenko D, Benes J, Pouska J, Lehmann C, Islam S, Cerny V (2019). Endothelial glycocalyx in acute care surgery - what anaesthesiologists need to know for clinical practice. BMC Anesthesiol.

[19] Chignalia AZ, Yetimakman F, Christiaans SC, Unal S, Bayrakci B, Wagener BM, Russell RT, Kerby JD, Pittet JF, Dull RO (2016). The glycocalyx and Trauma: A Review. Shock.

[20] Satchell SC, Tooke JE (2008). What is the mechanism of microalbuminuria in diabetes: a role for the glomerular endothelium?. Diabetologia.

[21] Ndisang JF (2018). Glomerular endothelium and its impact on glomerular filtration barrier in diabetes: are the gaps still illusive?. Curr Med Chem.

[22] Van Den Berg BM, Vink H, Spaan JAE (2003). The endothelial glycocalyx protects against myocardial edema. Circ Res.

[23] Okada H, Takemura G, Suzuki K, Oda K, Takada C, Hotta Y, Miyazaki N, Tsujimoto A, Muraki I, Ando Y, Zaikokuji R, Matsumoto A, Kitagaki H, Tamaoki Y, Usui T, Doi T, Yoshida T, Yoshida S, Ushikoshi H, Toyoda I, Ogura S (2017). Three-dimensional ultrastructure of capillary endothelial glycocalyx under normal and experimental endotoxemic conditions. Crit Care.

[24] Mukai S, Takaki T, Nagumo T, Sano M, Kang D, Takimoto M, Honda K (2021). Three-dimensional electron microscopy for endothelial glycocalyx observation using Alcian blue with silver enhancement. Med Mol Morphol.

[25] Takashima S, Tsuji S (2004). unique enzymatic properties of mouse sialyltransferases, ST6Gal II and ST8Sia VI. Trends Glycosci Glycotechnol.

[26] Takashima S, Tsuji S (2011). Functional diversity of mammalian sialyltransferases. Trends Glycosci Glycotechnol.

[27] Kitazume S, Imamaki R, Kurimoto A, Ogawa K, Kato M, Yamaguchi Y, Tanaka K, Ishida H, Ando H, Kiso M, Hashii N, Kawasaki N, Taniguchi N (2014). Interaction of platelet endothelial cell adhesion molecule (PECAM) with α2,6-sialylated glycan regulates its cell surface residency and anti-apoptotic role. J Biol Chem.

[28] Imamaki R, Ogawa K, Kizuka Y, Komi Y, Kojima S, Kotani N, Honke K, Honda T, Taniguchi N, Kitazume S (2018). Glycosylation controls cooperative PECAM-VEGFR2-β3 integrin functions at the endothelial surface for tumor angiogenesis. Oncogene.

[29] Dalziel M, McFarlane I, Axford JS (1999). Lectin analysis of human immunoglobulin G N-glycan sialylation. Glycoconj J.

[30] Schultz MJ, Swindall AF, Bellis SL (2012). Regulation of the metastatic cell phenotype by sialylated glycans. Cancer Metastasis Rev.

[31] Bhide GP, Colley KJ (2017). Sialylation of N-glycans: mechanism, cellular compartmentalization and function. Histochem Cell Biol.

[32] D'Addio M, Frey J, Otto VI (2020). The manifold roles of sialic acid for the biological functions of endothelial glycoproteins. Glycobiology.

[33] Betteridge KB, Arkill KP, Neal CR, Harper SJ, Foster RR, Satchell SC, Bates DO, Salmon AHJ (2017). Sialic acids regulate microvessel permeability, revealed by novel in vivo studies of endothelial glycocalyx structure and function. J Physiol.

[34] Lee C, Liu A, Miranda-Ribera A, Hyun SW, Lillehoj EP, Cross AS, Passaniti A, Grimm PR, Kim BY, Welling PA, Madri JA, DeLisser HM, Goldblum SE (2014). NEU1 sialidase regulates the sialylation state of CD31 and disrupts CD31-driven capillary-like tube formation in human lung microvascular endothelia. J Biol Chem.

[35] Chiodelli P, Rezzola S, Urbinati C, Signori FF, Monti E, Ronca R, Presta M, Rusnati M (2017). Contribution of vascular endothelial growth factor receptor-2 sialylation to the process of angiogenesis. Oncogene.

[36] Chandler KB, Leon DR, Kuang J, Meyer RD, Rahimi N, Costello CR (2019). N-Glycosylation regulates ligand-dependent activation and signaling of vascular endothelial growth factor receptor 2 (VEGFR2). J Biol Chem.

[37] Shibuya M (2013). Vascular endothelial growth factor and its receptor system: physiological functions in angiogenesis and pathological roles in various diseases. J Biochem.

[38] Sajina GC, Bellis SL, Hjelmeland AB (2022). ST6Gal1: oncogenic signaling pathways and targets. Front Mol Biosci.

[39] Cheng WK, Oon CE (2018). How glycosylation aids tumor angiogenesis: An updated review. Biomed Pharmacother.

[40] Obstfeld AE, Frey NV, Mansfield K, Lacey SF, June CH, Porter DL, Melenhorst JJ, Wasik MA (2017). Cytokine release syndrome associated with chimeric-antigen receptor T-cell therapy: clinicopathological insights. Blood.

[41] Gao YM, Xu G, Wang B, Liu BC (2021). Cytokine storm syndrome in coronavirus disease 2019: a narrative review. J Intern Med.

[42] Shirakura K, Ishiba R, Kashio T, Sakai M, Fukushima Y, Yamamoto N, Manabe S, Shigesada N, Tanaka T, Hino N, Aird WC, Doi T, Okada Y (2018). Endothelial Robo4 regulates IL-6 production by endothelial cells and monocytes via a crosstalk mechanism in inflammation. Biochem Biophys Res Commun.

[43] Simon M, Lücht C, Hosp I, Zhao H, Wu D, Heidecke H, Witowski J, Budde K, Riemekasten G, Catar R (2021). Autoantibodies from patients with scleroderma renal crisis promote PAR-1 receptor activation and IL-6 production in endothelial cells. Int J Mol Sci.

[44] Kang S, Tanaka T, Inoue H, Ono C, Hashimoto S, Kioi Y, Matsumoto H, Matsuura H, Matsubara T, Shimizu K, Ogura H, Matsuura Y, Kishimoto T (2020). IL-6 trans-signaling induces plasminogen activator inhibitor-1 from vascular endothelial cells in cytokine release syndrome. Proc Natl Acad Sci USA.

[45] McConnell MJ, Kawaguchi N, Kondo R, Sonzogni A, Licini L, Valle C, Bonaffini PA, Sironi S, Alessio MG, Previtali G, Seghezzi M, Zhang X, Lee AI, Pine AB, Chun HJ, Zhang X, Fernandez-Hernando C, Qing H, Wang A, Price C, Sun Z, Utsumi T, Hwa J, Strazzabosco M, Iwakiri Y (2021). Liver injury in COVID-19 and IL-6 trans-signaling-induced endotheliopathy. J Hepatol.

[46] Krick S, Helton ES, Easter M, Bollenbecker S, Denson R, Zaharias R, Cochran P, Vang S, Harris E, Wells JM, Barnes JW (2021). ST6GAL1 and α2-6 sialylation regulates IL-6 expression and secretion in chronic obstructive pulmonary disease. Front Immunol.

