# Supplementary material for: Gemcitabine alters sialic acid binding of the glycocalyx and induces inflammatory cytokine production in cultured endothelial cells
Source: Med Mol Morphol. 2023 Jan 9;56(2):128–37. doi: 10.1007/s00795-022-00347-4 (PMC9828377; doi:10.1007/s00795-022-00347-4)
Supplement: Supplementary file 1 — Supplementary file1 Figure S-1：Intracellular metabolic pathway of GEM and its associated enzymes After being up-taken into cells by equilibrative nucleoside transporter 1 (ENT1) and ENT2, GEM [2,2-difluorodeoxycytidine (dFdC)] is phosphorylated by deoxycytidine (dCK) into active nucleotide diphosphate (dFdCDP) and triphosphate (dFdCTP). These active nucleotides exhibit cell-killing activity by directly and indirectly inhibiting DNA synthesis. Directly, dFdCTP causes cell death (or apoptosis) after being incorporated into DNA strands by DNA polymerase in competition with deoxycytidine triphosphate (dCTP). Indirectly, dFdCDP inhibits RR (ribonucleotide reductase) to reduce intracellular dCTP concentration, thus indirectly enhancing DNA synthesis inhibition. dFdC, 2,2-difluorodeoxycytidine; ENT1 and ENT2, equilibrative nucleoside transporter 1 and 2; dCDA, deoxycytidine deaminase; dFdU, difluorodeoxyuridine; dCK, deoxycytidine kinase; 5’-NT, 5’-nucledtidase; dFdCMP, gemcitabine monophosphate; dCMPK, dCMP deaminase; dFdUMP, 2’2’-difluorodeoxyuridine monophosphate; dFdCDP, gemcitabine diphosphate; RR, ribonucleotide reductase; TS, thymidylate synthase; NDPK, nucleoside diphosphate kinase; dFdCTP, gemcitabine triphosphate (PPTX 40 KB) [file 795_2022_347_MOESM1_ESM.pptx]

## Slide 1
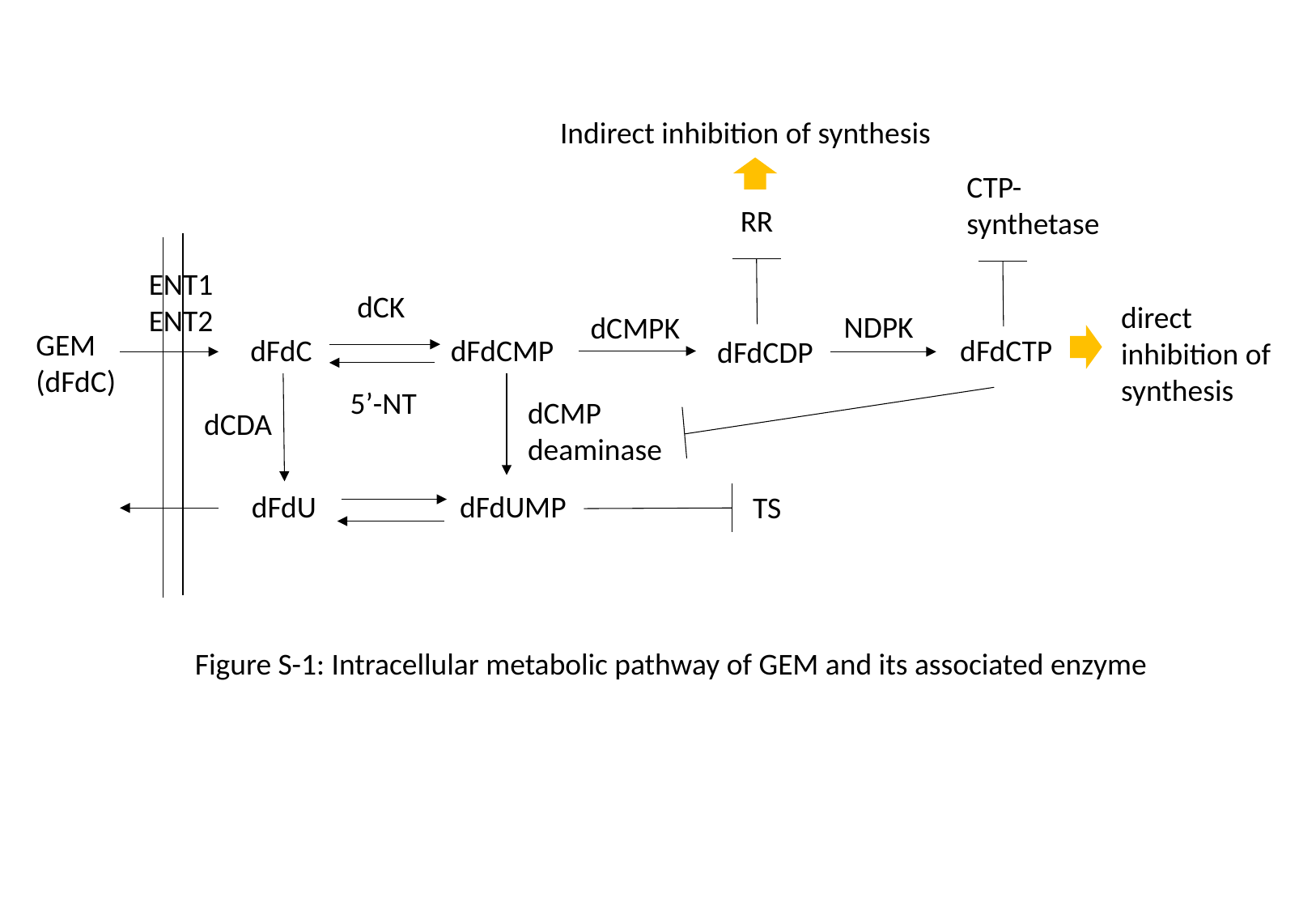

Indirect inhibition of synthesis
CTP-synthetase
RR
ENT1
ENT2
dCK
direct inhibition of synthesis
NDPK
dCMPK
GEM
(dFdC)
dFdC
dFdCMP
dFdCTP
dFdCDP
5’-NT
dCMP
deaminase
dCDA
dFdU
dFdUMP
TS
Figure S-1: Intracellular metabolic pathway of GEM and its associated enzyme
